# The effect of a clock’s presence on trauma resuscitation times in a Dutch level-1 trauma center: a pre–post cohort analysis

**DOI:** 10.1007/s00068-023-02371-0

**Published:** 2023-10-04

**Authors:** Eva Berkeveld, Kaoutar Azijli, Frank W. Bloemers, Georgios F. Giannakópoulos

**Affiliations:** 1 Department of Trauma Surgery, Amsterdam UMC Location Vrije Universiteit Amsterdam, De Boelelaan 1117, Amsterdam, The Netherlands; 2 Department of Emergency Medicine, Amsterdam UMC Location Vrije Universiteit Amsterdam, De Boelelaan 1117, Amsterdam, The Netherlands; 3 Department of Trauma Surgery, Amsterdam UMC Location University of Amsterdam, Meibergdreef 9, Amsterdam, The Netherlands

**Keywords:** Trauma, Time, Resuscitation, Clock, Trauma team

## Abstract

**Purpose:**

Interventions performed within the first hour after trauma increase survival rates. Literature showed that measuring times can optimize the trauma resuscitation process as time awareness potentially reduces acute care time. This study examined the effect of a digital clock placement on trauma resuscitation times in an academic level-1 trauma center.

**Methods:**

A prospective observational pre–post cohort analysis was conducted for six months before and after implementing a visible clock in the trauma resuscitation room, indicating the time passed since starting the in-hospital resuscitation process. Trauma patients (age ≥ 16) presented during weekdays between 9.00 AM and 9.00 PM were included. Time until diagnostics (X-Ray, FAST, or CT scan), time until therapeutic intervention, and total resuscitation time were measured manually with a stopwatch by a researcher in the trauma resuscitation room. Patient characteristics and information regarding trauma- and injury type were collected. Times before and after clock implementation were compared.

**Results:**

In total, 100 patients were included, 50 patients in each cohort. The median total resuscitation time (including CT scan) was 40.3 min (IQR 23.3) in the cohort without a clock compared to 44.3 (IQR 26.1) minutes in the cohort with a clock. The mean time until the first diagnostic and until the CT scan was 8.3 min (SD 3.1) and 25.5 min (SD 7.1) without a clock compared to 8.6 min (SD 6.5) and 26.6 min (SD 11.5) with a clock. Severely injured patients (Injury Severity Score (ISS) ≥ 16) showed a median resuscitation time in the cohort without a clock (*n* = 9) of 54.6 min (IQR 50.5) compared to 46.0 min (IQR 21.6) in the cohort with a clock (*n* = 8).

**Conclusion:**

This study found no significant reduction in trauma resuscitation time after clock placement. Nonetheless, the data represent a heterogeneous population, not excluding specific patient categories for whom literature has shown that a short time is essential, such as severely injured patients, might benefit from the presence of a trauma clock. Future research is recommended into resuscitation times of specific patient categories and practices to investigate time awareness.

## Introduction

Time and trauma are inextricably linked. Directly after traumatic injury, receiving adequate and timely care is essential [1–3]. The golden hour of trauma states that therapeutic interventions performed within the first hour after traumatic injury have the greatest effect on survival rates [4–6]. Therefore, providing swift and appropriate care in both the prehospital and in-hospital phases directly after trauma is crucial.

In the Dutch, mature, inclusive trauma system, prehospital care is provided by trained and experienced Emergency Medical Services (EMS) crews, if necessary, assisted by a Helicopter Emergency Medical Services (HEMS) crew [7]. A patient is subsequently triaged and transported to a level-1,-2, or -3 trauma center. A level-1 trauma center is equipped to provide care for severely injured patients (Injury Severity Score (ISS) ≥ 16), physiologic and hemodynamically unstable patients, and patients sustaining complex isolated injuries. Additional neurosurgical and cardiothoracic care is available on a 24-7 base [8]. Since the adagio treat first what kills first is crucial in trauma, patients are treated according to a protocolled decision tree, the Advanced Trauma Life Support (ATLS), during the resuscitation process in the Emergency Department (ED) [9]. A multidisciplinary trauma team in close collaboration provides the appropriate care as swiftly as possible in a horizontal approach, aiming to decrease the time until definitive care [10].

Insight into the duration of time-critical work is crucial to contribute to protocol development, education, and optimization of the process [11–13]. Previous studies identified trauma resuscitation times varying between 19.1 and 45.9 min for in-hospital trauma activations in various level-1 trauma centers [11, 14–16]. Besides, it was shown that the elapsed time as perceived by the healthcare professionals in time-critical work can differ from the actual time [17, 18]. Concerning trauma resuscitations in ED, Kuhlenschmidt identified that involved residents had a distorted perception of elapsed time, which was proportionate to the duration of the process [18].

The contributing effect of awareness of time to the process has been identified by Curtis et al. They showed a reduction in prehospital scene time for specific trauma patient categories after the HEMS crew was via audio made aware of the time elapsed [19]. Moreover, to facilitate time awareness, various time representation techniques, such as clocks showing the time elapsed since the start of the resuscitation, showed an appropriate option in trauma resuscitations [20]. Therefore, this study aimed to examine the effect of a clock’s presence on trauma resuscitation time. Additional insight into level-1 trauma center’s resuscitation times is obtained, and time durations of different phases of the resuscitation process will be evaluated.

## Methods

### Study setting

This study was conducted in level-1 trauma center Amsterdam UMC location VUmc. The trauma center is located in the trauma region of North West Netherlands and provides care for approximately 250 severely injured patients annually [21]. Trauma resuscitations in the Emergency Department (ED) are performed in one of two dedicated trauma resuscitation rooms, based on advanced triage criteria either a complete or selected trauma team is activated to provide the resuscitation [22]. During the day time, a complete team consists of a trauma surgeon, emergency physician, two ED nurses, anesthesiologist, nurse anesthetist, radiologist, two diagnostic radiographers, intensivist, and neurologist. During evening and night shifts, a surgical registrar and resident are present at the start of the resuscitation and the on-call trauma surgeon is present within 15 min. In comparison, a selective trauma team consists of an emergency physician, emergency resident, ED nurse, radiology resident, and diagnostic radiographer [8]. In both activated teams, ATLS-based trauma care is provided in close multidisciplinary collaboration [22].

### Study design and data collection

A prospective observational pre–post cohort analysis was conducted six months before and after implementing a visible clock in the trauma resuscitation room, indicating the absolute time and elapsed time since the start of the in-hospital resuscitation process. The measurements for cohort I, in which no clock was implemented, were performed between May 1, 2019, and August 1, 2019. In contrast, in cohort II, with the presence of a clock, patients between October 1, 2020, and January 1, 2021, were included. The sample size of 50 cases per cohort was chosen to detect a difference of at least half a standard deviation. All trauma patients (age ≥ 16) presented at the trauma resuscitation room during weekdays between 09.00 AM and 09.00 PM were included.

**Fig. 1:**
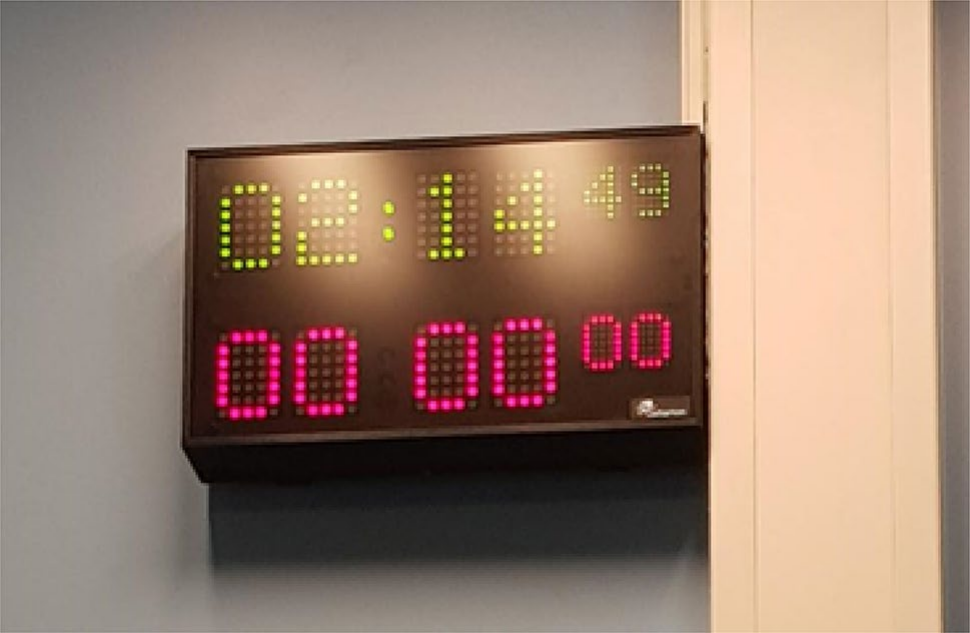
Trauma clock

As part of standard care, a visible clock was installed in each of the two trauma resuscitation rooms after the measurements of cohort I were completed. The clock contained digital information regarding the elapsed time from the start of the resuscitation process and the current time (Fig. 1). A familiarization period was introduced wherein all trauma team members could accustom themselves to the presence of the clock. The trauma team leader would start the clock at the moment of the patient’s arrival at the resuscitation room. After it was established that the team leader started the clock in over 90% of all trauma resuscitations, the inclusion period of cohort II commenced.

Data were collected by conducting measurements by trained researchers using a structured observation form. By no means interfered the researcher’s presence with the resuscitation process. Time until diagnostics (chest X-ray, FAST, or pelvic X-ray), time until CT scan, time until therapeutic intervention and total resuscitation time were measured manually with a stopwatch. Therapeutic interventions could be either one of the following: tracheal intubation, thoracotomy, thoracotomy, CPR, placement of a pelvic binder, placement of a second IV access, placement of an arterial line, placement of intraosseous access, administering a Mass Transfusion Protocol, reposition, wound treatment or suturing or placement of a bladder catheter. Patients and type of injury and mechanism characteristics were collected. The ISS was calculated based on the injuries classified by the Abbreviated Injury Score (AIS). Resuscitation times before and after trauma clock implementation were compared.

Total resuscitation time comprised the moment a patient entered the trauma resuscitation room on a stretcher until the definitive end of trauma resuscitation care. The ending of trauma resuscitation care concerned either the patient’s disposition from the trauma resuscitation room or when all resuscitative-related care was performed, and the patient was only still located there due to logistical reasons (e.g., a patient waiting for ICU or operation room availability).

After the inclusion period of cohort II was completed, a survey questionnaire was performed among trauma team leaders (i.e., trauma surgeons and emergency physicians) regarding their experience with the clock. Based on a five-point scale from strongly agree to strongly disagree, the following questions were surveyed, compatible with surveys from the current literature [23, 24].

1. The use of the clock is intuitive and easy to understand.
2. Overall, the clock makes resuscitation more time efficient.
3. The clock supports with timely disposition of patients with priority findings.
4. I am satisfied with the current use of a clock in trauma resuscitations.

### Data analysis

Continuous variables were presented as mean [standard deviation (SD)] or as median [interquartile range (IQR)] and were compared using independent sample *t*-tests or Mann–Whitney *U* tests. Categorical variables were described as frequencies and percentages and compared using Pearson’s chi-squared tests. Data were analyzed using IBM SPSS Statistics version 24.0 (IBM, New York, USA).

## Results

In total, 100 patients were included, 50 in each cohort. The total study population consisted predominantly of male patients (70.0%), with a median age of 48 years (SD 19.8) (Table 1). Blunt injury was the most common type of injury (91.0%), and a motor vehicle collision (34.0%) was the main caused mechanism of injury. Overall, the median ISS was 5.5 (IQR 8.3). For 51.0% of resuscitations, the complete trauma team was activated, whereas for 49.0% the selected trauma team was activated.

**Table 1:** Baseline characteristics

	Cohort I Without clock (*n* = 50)	Cohort II With clock (*n* = 50)	Total (*n* = 100)	*P* Value
Gender, male, *n* (%)	38 (76)	32 (64)	70 (70)	0.213
Age, years, mean (SD)	45.0 (19.4)	50.6 (20.0)	48.0 (19.8)	0.162
Comorbidity, *n* (%)				0.121
ASA 1	32 (64)	37 (74)	67 (67)	
ASA 2	9 (18)	13 (26)	22 (22)	
ASA 3	8 (16)	0	8 (8)	
ASA 4	1 (2)	0	1 (1)	
Type of injury, blunt, *n* (%)	46 (92)	45 (90)	91 (91)	0.347
Mechanism of injury				0.315
Motor vehicle collision	21 (42)	13 (26)	34 (34)	
Non-motorized traffic accident	11 (22)	8 (16)	19 (19)	
Shooting or stabbing incident	4 (8)	3 (6)	7 (7)	
Incident with a blunt object	1 (2)	4 (8)	5 (5)	
High energy fall	7 (14)	11 (22)	18 (18)	
Low energy fall	5 (10)	9 (18)	14 (14)	
Explosion	0	1 (2)	1 (1)	
Thermic accident	0	1 (2)	1 (1)	
Drowning	1 (2)	0	1 (1)	
ISS, median (IQR)	8.0 (9.3)	5.0 (8.3)	5.5 (8.3)	0.719
Trauma team, large, *n* (%)	27 (54)	24 (48)	51 (51)	0.548
Disposition after ED, *n* (%)				0.730
Own living environment	11 (22)	11 (22)	22 (22)	
Clinical admission	24 (48)	26 (52)	50 (50)	
ICU admission	6 (12)	6 (12)	12 (12)	
Operating room	6 (12)	3 (6)	9 (9)	
Inter-hospital transfer	2 (4)	4 (8)	6 (6)	
Death in ED	1 (2)	–	1 (1)	

*ASA* American Society of Anesthesiologists Classification; *ISS* Injury Severity Score; *ED* emergency department

**Fig. 2:**
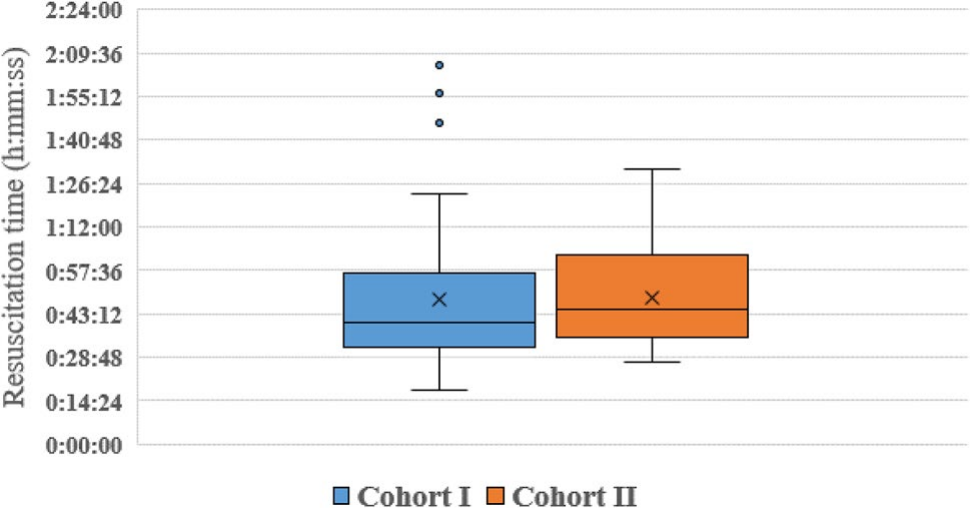
Resuscitation times per cohort

As illustrated in Table 2, the median total resuscitation time (including CT scan) was 40.3 min (IQR 23.3) in the cohort without a clock compared to 44.3 (IQR 26.1) minutes in the cohort with a clock. The observed variation in both cohorts is demonstrated in Fig. 2. The mean time until the first diagnostic and until the CT scan was 8.3 min (SD 3.1) and 25.5 min (SD 7.1) without a clock compared to 8.6 min (SD 6.5) and 26.6 min (SD 11.5) with a clock. In total, nine patients were considered severely injured (ISS ≥ 16) in the cohort without a clock and they showed a median resuscitation time of 54.6 min (IQR 50.5) (Table 3). In the cohort with a clock, eight patients were considered severely injured (ISS ≥ 16) with a median resuscitation time of 46.0 min (IQR 21.6).

**Table 2:** Duration characteristics

	Cohort I Without trauma clock (*n* = 50)	Cohort II With trauma clock (*n* = 50)	P value
Total resuscitation time, median (IQR)	40.3 (23.3)	44.3 (26.1)	0.970
Time until the first diagnostic, mean (SD)	8.3 (3.1) (*n* = 45)	8.6 (6.5) (*n* = 43)	0.637
Time until CT scan, mean (SD)	25.5 (7.1) (*n* = 43)	26.6 (11.5) (*n* = 45)	0.610
Time until first intervention, median (IQR)	9.4 (8.1) (*n* = 27)	13.5 (19.5) (*n* = 20)	0.589

Time in minutes

**Table 3:** Duration characteristics for severely injured patients (ISS ≥ 16)

	Cohort I Without trauma clock (*n* = 9)	Cohort II With trauma clock (*n* = 8)
Total resuscitation time, median (IQR)	54.6 (50.5)	46.0 (21.6)
Time until first diagnostic, mean (SD)	7.2 (2.5) (*n* = 8)	7.5 (1.3) (*n* = 8)
Time until CT-scan, mean (SD)	23.5 (3.1) (*n* = 7)	20.1 (3.4) (*n* = 8)
Time until first intervention, median (IQR)	08.2 (11.1) (*n* = 7)	12.1 (35.3) (*n* = 5)

Time in minutes

In the cohort without a clock, two patients received cardiopulmonary resuscitation in ED of whom one also underwent an emergency laparotomy in the OR, one patient required tracheal intubation in ED, and one patient required an emergent craniotomy in the OR directly after resuscitation in ED.

In the cohort with a clock, one patient required tracheal intubation, one patient required a thoracostomy, two patients were after resuscitation in ED transferred to the specialized burn center, one patient required an emergent craniotomy in the OR, and two patients required emergent spinal decompressive surgery in the OR directly after their resuscitation in ED.

Furthermore, the survey was sent out to 12 trauma team leaders, of whom 10 responded. Forty percent (*n* = 4) of respondents agreed that the clock made the resuscitation more time efficient, whereas 40% (*n* = 4) were neutral, 10% (*n* = 1) strongly agreed, and 10% (*n* = 1) disagreed (Table 4). With the statement that the clock supports the timely disposition of patients with priority findings, 50% (*n* = 5) of respondents were neutral, 20% (*n* = 2) agreed, 10% (*n* = 1) strongly agreed, and 20% (*n* = 2) disagreed.

**Table 4:** Survey questionnaire

	Strongly agree	Agree	Neutral	Disagree	Strongly disagree
The use of the clock is intuitive and easy to understand	5 (50%)	5 (50%)	–	–	–
Overall, the clock makes resuscitation more time efficient	1 (10%)	4 (40%)	4 (40%)	1 (10%)	–
The clock supports with timely disposition of patients with priority findings	1 (10%)	2 (20%)	5 (50%)	2 (20%)	–
I am satisfied with the current use of the clock in trauma resuscitations	1 (10%)	2 (20%)	3 (30%)	4 (40%)	–

## Discussion

This study examined the effect of a clock on trauma resuscitation times in a Dutch level-1 trauma center. Based on data in this study, no effect on resuscitation time was found after clock placement. The fact that we found no evidence for an association could be explained by several factors.

In trauma, a short time until definitive care is essential [1–6, 25]. In the (inter-)national literature, short time was found to benefit patients’ survival in the case of hemodynamically unstable trauma patients [1, 4, 25, 26], especially when concurrently suffering from Traumatic Brain Injury (TBI) [5]. Therefore, it might be that during trauma resuscitation, a short time is more vital for patients with specific types of injury, such as TBI, severe hemorrhage, or severely injured patients (ISS ≥ 16). This current study was characterized by a heterogeneous study population, consisting of patients with various severities of injury, mechanism of injury, and anticipated required trauma team (i.e., complete or selected) as triaged based on the prehospital situation report by (H)EMS. Likewise, experience from the prehospital setting in a previous study by Curtis et al. showed that awareness of time can reduce scene time for specific patient categories (i.e., patients with a prehospital GCS below eight or patients requiring anesthesia) [19]. Therefore, it might be that for some patient categories, for whom we know the time to definitive care is crucial, a time awareness intervention through a clock might have a greater effect than for others. It is thus possible that a clock’s presence in the trauma resuscitation room is less relevant for time duration within the rather heterogeneous population, as observed in our study.

In our study, the clock was implemented as a form of standard care practice. Rigorous efforts were in place for the trauma team to familiarize with the presence of the clock and the team leader to activate the elapsing time the moment the patient entered the resuscitation room. The observational design left the actual use of the clock’s information up to the professional team member’s expertise. However, it might have been that the current implementation did not properly support time awareness within the trauma teams. For half of the team leaders, awareness of the elapsed time did occur (50%), whereas neutrality (40%) or absence (10%) were less frequent. In comparison, Curtis et al., examining the effect of a prehospital audible scene timer, found a vast majority (91.0%) of the HEMS crew aware of the elapsed time. However, in 57.0%, no perceived change in practice was experienced by the crew, while they did find a time reduction for specific patient categories [19]. Perhaps room for improvement of the clock’s utilization exists, as for the minority (30%) the clock was used to their satisfaction during the resuscitation. The placement location on the patient’s left-hand side contributed to a clear view for the clinician performing the primary survey and a rather easy view for the trauma team leader. However, the placement of a visual time indicator near the vital signs monitors, similar to Kusunoki et al., might benefit awareness due to the already allocated attention to this information source [20].

Furthermore, it might have been that other factors during the trauma resuscitation have had a more substantial influence on the resuscitation’s duration than the clock alone. In the acute settings of trauma resuscitations, team structure and collaboration are essential, as team functioning and organization errors can lead to significantly more alteration in treatment [11]. Additionally, leadership attributes play a crucial role in the resuscitation process [10]. Previous studies showed that the presence of an attending trauma surgeon upon patients’ arrival could significantly reduce the time till diagnostics [27, 28] and total resuscitation time [29, 30]. Moreover, the degree of experience of the trauma team leader has a major impact on process functioning [31, 32], with an empowering leadership style being more appropriate in situations with less severely injured patients or an experienced team [32].

The total resuscitation time in this study is rather similar to some of the previous studies conducted in Dutch level-1 trauma centers [14, 16]. Spanjersberg et al. found median resuscitation times of 45.9 and 34.8 min for patients suffering from blunt injury and resuscitated by the “minor” versus “major” trauma team, respectively. The mean ISS of patients assessed by the “minor” team was 7.0, whereas, for patients assessed by the “major” team, it was 22.0 [14]. Van Olden et al. showed, similar to our results, resuscitation times with medians of 41.3 and 44.0 min for severely injured patients (ISS ≥ 16) receiving prehospital HEMS and EMS care versus EMS care alone, respectively [16].

In comparison, in line with a reached consensus in a Delphi study by Hoogervorst et al. who advocated for a maximum ED resuscitation time of 30 min [33], some studies observed a relatively shorter resuscitation time than ours. Lubbert et al., found a median of 32.9 min [11]. Van Maarseveen et al. found mean resuscitation times of 23.4 and 19.1 min for severely injured patients (ISS ≥ 16) resuscitated in a setting with an on-call versus in-house trauma surgeon, respectively [15]. These shorter resuscitation times compared to our findings might plausibly be caused by differences in the severity of the included patients, as our study’s population median ISS of 5.5 (8.3) was relatively low. Previous recent studies conducted in Dutch level-1 trauma centers showed that in their resuscitated population, the shorter the resuscitation time, the higher the severity of the injury was [14, 30].

In this study, a strengthening factor was the prospective nature of the design. Moreover, as far as we know, this was the first study examining the effect of a trauma clock on resuscitation times. A limiting factor was the small group sizes. The observed variation of resuscitation times was larger than expected, which might have contributed to the non-significant findings. Furthermore, a certain degree of bias caused by the two different inclusion periods between the cohorts cannot be excluded. Influences of COVID-19, which existed at the start of the inclusion period of the cohort with trauma clock, cannot be ruled out. However, the cohort with a clock did not consist of (suspected) COVID-19-positive patients, so no influence of COVID-19 measurements on trauma resuscitation time is expected. Consistent with peak hours in patient influx in the literature, the inclusion occurred from 09.00 AM until 09.00 PM. However, this limits the generalizability of a trauma clock’s effect on resuscitation time outside these hours. Interpersonal variability was limited due to the use of structured observation forms and identical training of the observers. Due to the equal presence of a researcher in the trauma resuscitation room in both cohorts, we believe a possible Hawthorne effect, at which a team functions differently because they are being observed, would be equally negated between both cohorts. To minimize the possible effects, the researcher was positioned behind a window wall, not involved in the resuscitation process. The heterogeneous study population, in line with the general trauma population, contributes to the generalizability of the study. Future research into resuscitation times of specific patient categories and practices is recommended to investigate time awareness.

## Conclusion

This study found no effect on resuscitation time after clock placement in the trauma resuscitation room of a Dutch level-1 trauma center for patients with potentially life-threatening injuries. Nonetheless, the data represent a heterogeneous population, and it does not exclude specific patient categories for whom literature has shown that a short time is essential, such as severely injured patients, who might benefit from the presence of a trauma clock. Future research is recommended into resuscitation times of specific patient categories and practices to investigate time awareness.

## Data Availability

Original data remain available and access may be provided upon reasonable request.
